# Delineating the Role of Mitophagy Inducers for Alzheimer Disease Patients

**DOI:** 10.14336/AD.2020.0913

**Published:** 2021-06-01

**Authors:** Wen-Wen Wang, Ruiyu Han, Hai-Jun He, Zhen Wang, Xiao-Qian Luan, Jia Li, Liang Feng, Si-Yan Chen, Yahyah Aman, Cheng-Long Xie

**Affiliations:** ^1^The center of Traditional Chinese Medicine, The Second Affiliated Hospital and Yuying Children's Hospital of Wenzhou Medical University, 325027, Wenzhou, China.; ^2^Department of Neurology, The First Affiliated Hospital of Wenzhou Medical University, Wenzhou 325000, China.; ^3^NHC Key Laboratory of Family Planning and Healthy, Hebei Key Laboratory of Reproductive Medicine, Hebei Research Institute for Family Planning Science and Technology, Shijiazhuang, Hebei 050071, China.; ^4^Department of Clinical Molecular Biology, University of Oslo, Akershus University Hospital, Lørenskog, Norway.

**Keywords:** mitophagy, Alzheimer’s disease, deep learning, mitophagy inducers, systematic review

## Abstract

Alzheimer’s disease (AD) is the most common cause of dementia in elderly that serves to be a formidable socio-economic and healthcare challenge in the 21^st^ century. Mitochondrial dysfunction and impairment of mitochondrial-specific autophagy, namely mitophagy, have emerged as important components of the cellular processes contributing to the development of AD pathologies, namely amyloid-β plaques (Aβ) and neurofibrillary tangles (NFT). Here, we highlight the recent advances in the association between impaired mitophagy and AD, as well as delineate the potential underlying mechanisms. Furthermore, we conduct a systematic review the current status of mitophagy modulators and analyzed their relevant mechanisms, evaluating on their advantages, limitations and current applications in clinical trials for AD patients. Finally, we describe how deep learning may be a promising method to rapid and efficient discovery of mitophagy inducers as well as general guidance for the workflow of the process.

## Introduction

Alzheimer’s disease (AD) is an age-related and progressive neurodegenerative disorder that is the most common cause of dementia affecting over 45 million people worldwide [1]. It is clinically characterized by progressive loss of memory, cognitive impairment, social and occupational dysfunction [2, 3]. The neuropathological hallmarks of AD are extracellular senile plaques, composed of accumulation of amyloid-β (Aβ), and intracellular neurofibrillary tangle (NFT), containing aberrantly hyper-phosphorylated microtubule associated protein tau (MAPT/p-tau) [4, 5]. These hallmarks are accompanied by neuroinflammation [6], vascular dysfunction [7], genomic instability/ApoE4 [8], aberrant neuronal activity [5], synaptic loss and dysfunction [3], cell senescence, impaired DNA repair [9], comprised autophagy [10] and mitochondrial dysfunction [11]. Despite decades of extensive research, current therapeutic strategies are symptomatic and do not halt or slow the progression of the disease. Over the past 15 years over 250 drug candidates have been attempted for potential treatment AD, largely targeting the proteins p-tau or Aβ, but all have been unsuccessful [12]. This can partially be explained by the heterogeneity of the disease as well as an incomplete understanding of the fundamental cause of AD [13]. Therefore, it is important to understand the intricate molecular mechanisms underlying the neurodegenerative processes in AD that may provide novel therapeutic interventions to halt the progression of the disease.

Increasing body of evidence suggests that the accumulation of dysfunctional mitochondria is a common feature of both sporadic and familial AD patients, as well as experimental models of AD [11, 14-16]. It has been proposed that inefficient clearance of damaged mitochondria through a selective form of autophagy, namely mitophagy, results in a viscous cycle that seeds the development and propagation of AD pathology [11, 17]. Thus, in this review we will highlight the mitophagy impairment in AD as well as evaluate the therapeutic potential of promoting mitophagy as a strategy against AD progression.

### Mechanism of mitophagy

Mitochondria are the “powerhouse” of cells that perform various critical roles in cellular homeostasis and are involved in regulating various aspects of cell function that include: oxidative phosphorylation, calcium (Ca^2+^) homeostasis in synapse, metabolism and biosynthesis of intermediates for cell growth or death [18, 19]. Dysfunction of mitochondria can be fatal for cellular bioenergetic and metabolic requirements and therefore can result is development of a spectrum of disorders, including AD [20]. In order to maintain mitochondrial homeostasis, a highly evolutionary conserved quality-control system termed as mitophagy is in place, to clear damaged and/or superfluous mitochondria. The process of mitophagy plays a crucial role in mitochondrial and metabolic homeostasis, energy supply, neuronal survival, and health [21, 22]. Several mitophagy-mediating pathways have been identified, many of which are conserved from *C. elegans* to humans [23-26]. The following section describes recent advancements in the understanding of basic molecular mechanisms that mediate mitophagy.

The PINK1-Parkin pathway is one of the well-described pathways mediating mitophagy that is triggered upon mitochondrial membrane depolarization. Under physiological conditions, phosphatase and tensin homolog (PTEN)-induced putative kinase 1 (PINK1), a serine/threonine kinase, is transported into the inner mitochondrial membrane (IMM), where it is processed and cleaved by several proteases [27]. However, in response to stress (i.e. oxidative, and starvation), PINK1 is stabilized on the outer mitochondrial membrane (OMM) [28]. On the OMM, PINK1 is activated by auto-phosphorylation and subsequently phosphorylates mitofusin 2 (MFN2) and ubiquitin, which induce the recruitment of parkin, an E3-ubiquitin ligase, to the OMM surface. PINK1-induced phosphorylation alters parkin conformation, promoting its association with the mitochondrial surface and triggering its E3 ligase activity [29]. Phosphorylated poly-ubiquitin chains serving as an “swallow me” signal for the autophagic machinery [30]. Meanwhile, parkin ubiquitinates several OMM proteins that are in turn recognized by the adaptor proteins ubiquitin-binding proteins optineurin (OPTN), p62, nuclear dot protein 52 (NDP52) and neighbor of BRCA1 (NBR1) [31], which recruit the assembling autophagosomes to the cargoes through binding to microtubule-associated protein 1A/1B-light chain 3 (MAP1LC3) [32]. In addition to Parkin, Gp78, SMURF1, MUL1, SIAH1 and ARIH1 represent alternative E3 ubiquitin ligases targeting OMM proteins prior to mitophagy. The PINK1-Parkin pathway modulates mitochondrial dynamics and motility by targeting MFN and Miro for proteasomal degradation [33].

**Figure 1.**
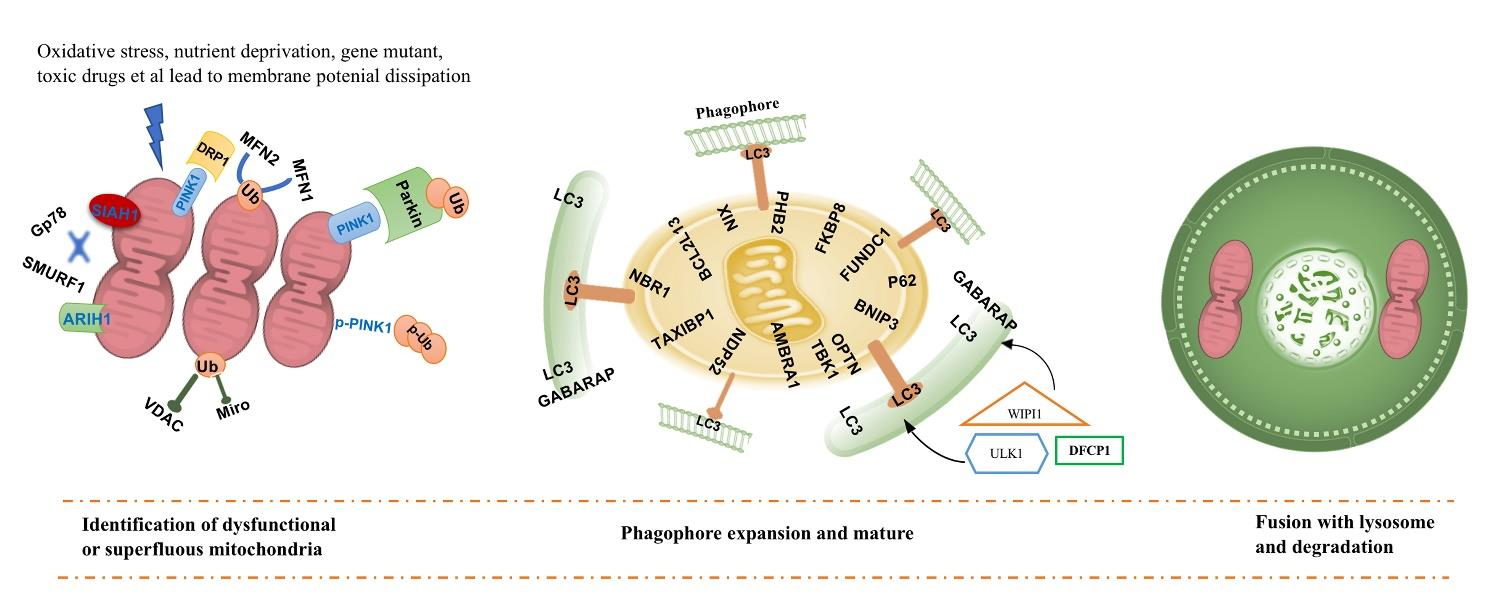
Schematic of mitophagy machinery: Mitophagy pathway comprises identification of dysfunctional or superfluous mitochondria, Phagophore expansion and mature, fusion with the lysosome, and finally, degradation. Reduced mitochondrial membrane potential enables the stabilization of PINK1 at the OMM. PINK1 is activated by auto-phosphorylation and then phosphorylates MFN2 and ubiquitin, giving rise to recruit Parkin to the OMM surface. Phosphorylated poly-ub chains serving as an “swallow me” signal for the autophagic machinery. Meanwhile, parkin ubiquitylates several outer membrane components that are following recognized by the adaptor proteins ubiquitin-binding proteins OPTN, p62, NDP52 and NBR1, which recruit the impaired mitochondria to the autophagy pathway and initiate autophagosome formation through binding with LC3 (this pathway refers to PINK1-Parkin mediate mitophagy). In addition, BNIP3, NIX and FUNDC1, PHB2 and cardiolipin et al, as mitophagy receptors, localize to the OMM and connect directly with LC3 following mitochondrial impairment to induce mitochondrial elimination. Different receptors ensure specificity of the process in different tissues and following diverse stimuli (PINK1-Parkin independent mitophagy).

In addition to PINK1-dependent mitophagy, mitochondrial proteins serve as mitophagy receptors, targeting dysfunctional mitochondria directly to autophagosomes for degradation. Mitophagy receptors interact directly with LC3 via the LIR (LC3-interacting region) and/or gamma aminobutyric acid type A receptor-associated protein (GABARAP) autophagosomal membrane proteins, to mediate mitochondrial elimination. Thus, representing a form of of PINK1-Parkin-independent mitophagy [34]. Among the mitophagy receptors identified include OMM proteins such as BNIP3, NIX, FUNDC1, AMBRA1, and MUL1, as well as IMM proteins cardiolipin and PHB2. The IMM proteins needs proteasome-dependent rupture of the OMM in order to couple damaged mitochondria with LC3 phagophore following mitochondrial impairment to induce mitochondrial elimination [31, 35]. A schematic summary of the mitophagy machinery in Figure 1.

## Defective mitophagy in AD

An intro into defective mitophagy in AD will be useful before the crosstalk between AD pathology and mitophagy impairments.

### Crosstalk with Aβ

Mitochondrial dysfunction and a bioenergetic deficit may contribute to the devolvement of AD-associated Aβ plaques. Conversely, Aβ deposition can also interact and contribute to deterioration of mitochondrial homeostasis, thus giving rise to a “vicious cycle” [36]. Evidence stemming from mammalian cells and human post-mortem brain specimens from AD patients have demonstrated that mitochondrial dysfunction is a characteristic feature in AD and is associated with the development and progression of Aβ deposits via oxidative stress [37]. In fact, administration of superoxide dismutase-2 (SOD2) was shown to protect against hAPP/Aβ-induced impairments in the aging brain with improvement of mitochondrial function highlighted as a mechanism of action [38]. Accumulation of unrepaired damaged nuclear due to dysfunction of base excision repair and DNA double-strand break repair and mitochondrial DNA may occur early in the AD, leading to Aβ increase by inhibition of the nicotinamide adenine dinucleotide (NAD^+^)/sirtuin-PGC-1a pathway [9]. Moreover, a growing body of evidence reports that Aβ peptides exerts a detrimental effect on mitochondrial function. In particular, accumulation of Aβ in neurons that harbor mutantation in the amyloid precursor protein (APP) leads to reduction of mitochondrial ATP production, decresaed mitochondrial membrane potential, and enzymes activity, coupled to increasing levels of mitochondrial reactive oxygen species (ROS) [39]. Furthermore, evidence in animal models of AD including *C. elegans* expressing human Aβ_1-42_ pan neuronally and transgenic APP/PS1 mice display development and progression of memory deficits associated with defective mitophagy [11]. Inhibition of mitochondrial permeability transition pores and reduction in mitochondrial injury or inducing mitophagy through small molecule mitophagy inducers (e.g. NMN: nicotinamide mononucleotide, UA: urolithin A or NR: Nicotinamide riboside) were demonstrated to protect neurons against the Aβ neurotoxicity [11, 40]. In addition, recent studies indicate a close link of disrupted-in-schizophrenia-1 (DISC1) to AD pathogenesis. DISC1, an LC3-binding mitophagy protein, has been shown to be reduced in Aβ-treated mammalian cells, AD patients post-mortem brain samples and in the transgenic APP/PS1 mice. In fact, Aβ-induced mitochondrial dysfunction, loss of spines, impaired synaptic plasticity and impaired long-term potentiation (LTP) were rescued upon DISC1 overexpression in the APP/PS1 mice [41]. In addition, overexpression of DISC1 enhances mitophagy through its binding to LC3, whereas knockdown of DISC1 blocks Aβ-induced mitophagy [41].

### Crosstalk with Tau

Accumulating evidence stemming from AD human post-mortem tissue as well as models of AD, namely drosophila, murine and human cell lines overexpressing wild-type (WT) and/or mutant tau implicate impairments in mitochondrial morphology, function, dynamics, and transport [17, 40, 42]. In particular, mitochondrial localization of p-tau has been associated with impairments in mitochondrial Ca^2+^ homeostasis, mitochondrial-ER communication, and mitophagy [43]. Further evidence from *C. elegan*s, murine and human cell lines overexpressing WT and/or mutant tau implicate impaired mitophagy by reduced phosphorylation of mitophagy initiation proteins, namely ULK1 and TBK1, which leads to accumulation of damaged mitochondria and functionally to cognitive deficits [11, 17]. Moreover, small compounds UA and actinonin (AC), mitophagy inducers, can improve the mitochondria homeostasis and mitophagy levels, as well inhibited multiple tau phosphorylation sites in the hippocampi of 3xTgAD mice [11]. Altogether, implicating the beneficial effect of boosting mitophagy in AD that can be considered as a viable therapeutic strategy, which can slow the development and progression of the disease affecting over 45 million people worldwide.

**Figure 2.**
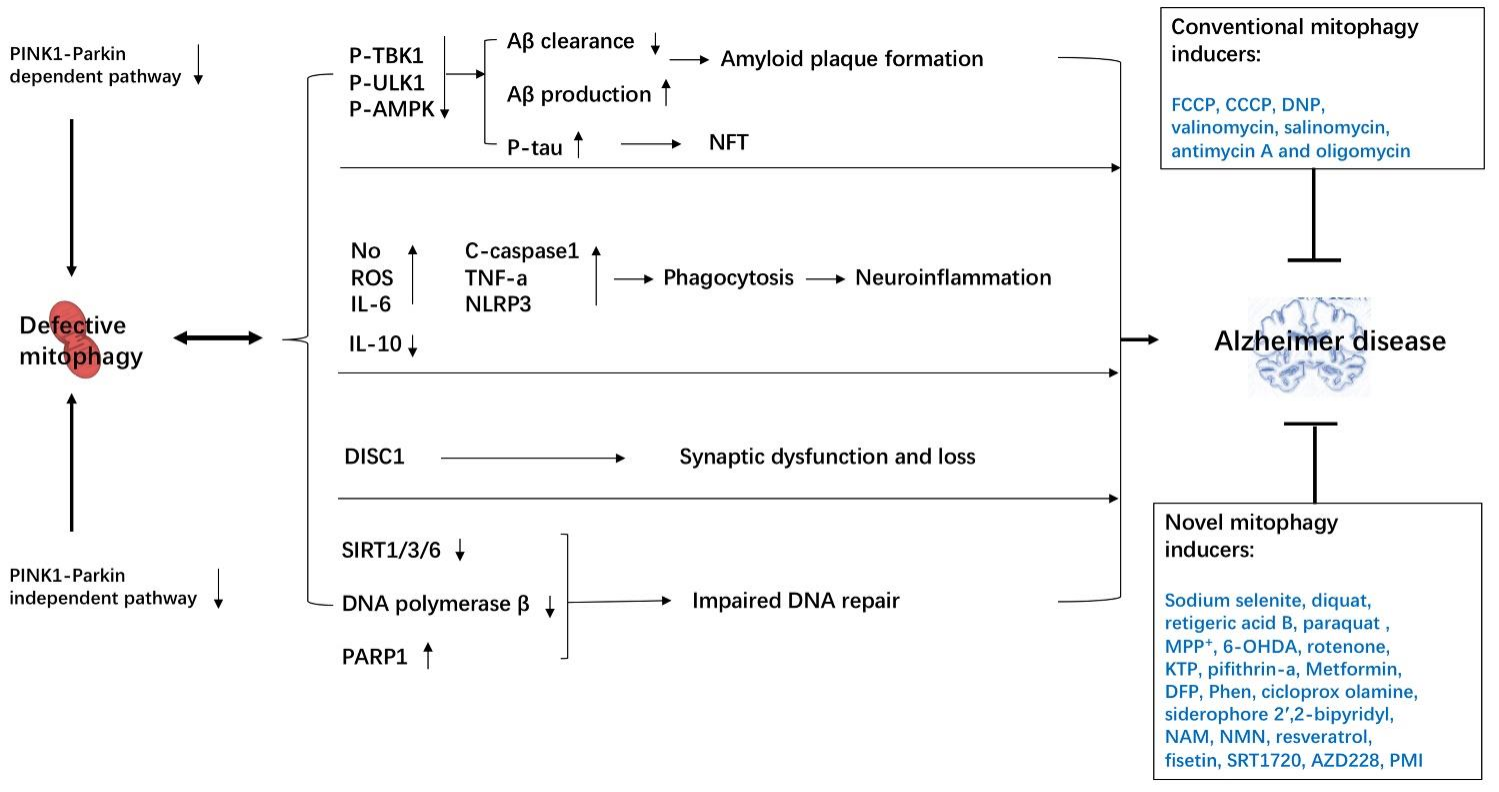
Mechanisms on how mitophagy links to AD: Molecular pathways of mitophagy, including PINK1-Parkin-dependent or -independent pathway. Defective mitophagy results in accumulation of Aβ deposits, neurofibrillary tangles, neuroinflammation, synaptic dysfunction and impair DNA repair ability through multiple molecular mechanisms, all of which contribute to AD pathogenesis. Moreover, restoration of mitophagy by mitophagy inducers is likely to be crucial to against AD.

Moreover, in AD models, several preclinical studies have shown that immune activation, including microglia and several cytokines, has the capacity to trigger and drive the pathophysiology of AD [6]. Defective mitophagy also involves in the neuroinflammation. There is a strong inflammatory phenotype in both *Pink1*^-/-^ and *Parkin^-/-^* mice, both of which were central regulators in the mitophagy molecular mechanism [44]. Additionally, restoration of neuronal mitophagy through treatment with UA or AC reduced Aβ burden via increased engulfment of plaques by microglia and interleukin-10 (IL-10) level, as well as decreased brain levels of key pro-inflammatory cytokines including IL-6, cleaved-caspase1, NLRP3 inflammasome, and tumour necrosis factor (TNF) [11]. Recently, one paper showed that in xeroderma pigmentosum group A (XPA), a nucleotide excision DNA repair disorder with severe neurodegeneration, existed defective mitophagy. The mitochondrial abnormalities appear to be caused by decreased activation of the NAD^+^-SIRT1-PGC-1a axis triggered by hyperactivation of the DNA damage sensor PARP-1. This phenotype is rescued by PARP-1 inhibition or by supplementation with NAD^+^ precursors [45]. NAD^+^ ameliorates Ataxia Telangiectasia Models phenotypes through upregulation of mitophagy and DNA repair [46]. Furthermore, NR-treated 3xTgAD/Polβ^+/-^ mice exhibited reduced DNA damage, neuroinflammation, and increased the DNA repair and activity of SIRT3 in the brain [47]. Summary the mechanisms of how mitophagy and its impairment is associated with the pathogenesis of AD in displayed Figure 2.

## Mitophagy inducers as a therapeutic intervention for AD models

Due to impaired mitophagy is common in AD, and maybe a causal mechanism, restoring mitophagy may provide to be a therapeutic strategy for AD. Recently, one comprehensive paper found that restoration of neuronal mitophagy by NAD^+^ precursor supplementation (NMN, UA and AC), can reduce insoluble Aβ_1-42_ and Aβ_1-40_ levels and against cognitive impairment in both *C. elegans* and APP/PS1 AD mouse model through microglial phagocytosis and suppression of neuroinflammation. Thus, suggesting that impaired removal of damaged mitochondria is critical in the development ad progression of AD [11]. Moreover, treatment with NR improved memory acquisition and retention as well as inhibited AD-associated p-tau pathology, reduced DNA damage and restored hippocampal synaptic plasticity in transgenic AD model [47]. Moreover, treatment of 3xTgAD mice with nicotinamide (NAM) for 8 months improves cognitive performance, reduces Aβ and p-tau pathologies in cerebral by a mechanism involving NAD^+^ biosynthesis [48]. In addition to NAD^+^ precursor relevant mitophagy inducers, several energy modulators, including metformin, pifithrin-a, resveratrol, spermidine, p62-mediated mitophagy inducer (PMI), UA and AC, have been shown to maintain mitochondrial integrity and boost mitochondrial biogenesis through mitophagy induction [25, 49]. Additional pharmacological compounds that are toxic mitophagy inducers, mainly leading to mitochondria damage also can induce mitophagy activity, such as CCCP, FCCP, 2,4-dinitrophenol (DNP), 1-methyl-4-phenylpyridinium (MPP+), 6-hydroxyldopamine (6-OHDA), and sodium selenite [49]. We have summarized the small molecule compounds inducing mitophagy and their chemical structure as well relevant mechanism in the Table 1.

**Table 1:** List of the small molecule compounds inducing mitophagy.

Mitophagy inducers	Chemical formula	Mechanism related to mitophagy	AD clinical trial (Yes/No)	Ref
Non-toxic				
kinetin triphosphate (KTP)	C10¹³C5H20N5O14P3	Be able to activate PINK1	No	[72]
Pifithrin-a	C_16_H_19_BrN_2_OS	A specific inhibitor of p53, ameliorates mitochondrial dysfunction and preserves Parkin-mediated mitophagy	No	[73]
Deferiprone (DFP)	C_7_H_9_NO_2_	Iron chelator makes loss of iron triggers PINK1/Parkin-independent mitophagy	No	[74]
Metformin	C_4_H_11_N_5_	Restores Parkin-Mediated Mitophagy, Suppressed by Cytosolic p53 and inducing AMPK, sirtuin et al	Yes	[23, 75]
1,10'-phenanthroline (Phen)	C_12_H_8_N_2_	Mitochondrial fragmentation and fission caused by phenanthroline promotes mitophagy	No	[76]
Ciclopirox olamine	C_12_H_17_NO_2_	Dissipation of the mitochondrial membrane potential (ΔΨm) and stimulate p21 expression	No	[77]
Nicotinamide riboside (NR)	C_11_H_15_N_2_O_5_+	NAD+ accumulation: activation of SIRT1 and induces mitochondrial fission	No	[11]
Nicotinamide (NAM)	C_6_H_6_N_2_O	Elevation of NAD+/NADH ratio may promote cellular health by facilitating mitochondrial autophagy	No	[78]
Nicotinamide mononucleotide (NMN)	C_11_H_15_N_2_O_8_P	Increased PINK-1, PDR-1, or DCT-1-dependent pathways	Yes	[11]
Resveratrol:	C_14_H_12_O_3_	Activation of AMPK and SIRT et al	Yes	[79]
Fisetin	C_15_H_10_O_6_	Activation of SIRT	No	[80]
P62/SQSTM1-mediated mitophagy inducer (PMI)	C_14_H_9_IN_4_O_2_	Without recruiting Parkin or collapsing ΔΨm and P62/SQSTM1-dependent	No	[81]
Spermidine	C_7_H_19_N_3_	Enhanced mitophagy and mitochondrial respiration dependent Atg5	No	[82]
Urolithin A	C_13_H_8_O_4_	Increased PINK-1, PDR-1, or DCT-1-dependent pathways	No	[83]
Actinonin	C_19_H_35_N_3_O_5_	Enhanced kinase activity of PINK1 and promote Mitochondrial fission	No	[84]
Rapamycin	C_51_H_79_NO_13_	Increasing the translocation of p62 and Parkin to the damaged mitochondria	No	[85]
Toxic				
Phenylhydrazones carbonyl cyanide m-chlorophenyl hydrazone (CCCP)	C_9_H_5_ClN_4_ C_10_H_5_F_3_N_4_O_5_ FCCP: R1=H; R2=OCF3 CCCP: R1=CI; R2=H	Proton-leak-induced loss ofΔΨm in AMPK-independent pathway	No	[86]
Carbonyl cyanide-p-(triuoromethoxy) phenylhydrazone (FCCP)			No	[87]
2,4-dinitrophenol (DNP)	C_6_H_4_N_2_O_5_	Dissipation of the ΔΨm	No	[88]
BAM15	C1_6_H_10_F_2_N_6_O	Dissipation of the ΔΨm	No	[89]
Valinomycin	C_54_H_90_N_6_O_18_	ΔΨm collapse due to K+ influx	No	[90]
Salinomycin	C_42_H_70_O_11_	Induce mitophagy, mitoptosis and increased ∆Ψ and reduced ATP level	No	[91]
Antimycin A	C_28_H_40_N_2_O_9_	Increased superoxide generation coupled with ∆Ψ loss	No	[92]
Oligomycin	C_28_H_40_F_3_N_2_O_9_		No	
Sodium selenite	Na_2_O_3_Se	MUL1, a mitochondria-localized E3 ligase, regulates selenite-induced mitophagy in an ATG5 and ULK1-dependent manner	Yes	[93]
Diquat	C_12_H_12_N_2_Br_2_	Diquat-induced oxidative stress increases, impairs mitochondrial function, and triggers mitophagy	No	[94]
Retigeric acid B (RAB)	C_30_H_46_O	RAB induces mitochondrial damage and mitophagy	No	[95]
Paraquat	C_12_H_14_Cl_2_N_2_	Superoxide-induced mitochondrial damage	No	[96]
Rotenone	C_23_H_22_O_6_	Externalization of cardiolipin acting as the signal to remove damaged mitochondria	No	[97]
1-methyl-4-phenylpyridinium (MPP+)	C_12_H_12_N^+^	ROS accumulation and mitochondrial damage: ERK1/2-dependent mitophagy	No	[98]
6-hydroxyldopamine (6-OHDA)	C_8_H_11_NO_3_	Superoxide-induced mitochondrial damage	No	[99]

PINK1: Phosphatase and tensin homolog (PTEN)-induced kinase 1; AMPK: Adenosine 5’-monophosphate-activated protein kinase; SIRT1: Sirtuin 1; NAD+: nicotinamide adenine dinucleotide; NADH: Nicotinamide adenine dinucleotide; PDR-1: pectin degradation regulator-1; DCT-1/SLC11A2: solute carrier family 11 member 2; P62/SQSTM1: heat shock 90-like protein; MUL1: mitochondrial E3 ubiquitin protein ligase 1; ATG5: autophagy related 5; ULK1: unc-51 like autophagy activating kinase mitochondrial; RAB: Retigeric acid B; ROS: Reactive oxygen species; Erk: extracellular signal-regulated kinase.

## Systematic review of mitophagy inducers in clinical trials for AD patients

We conducted a systematic search on PubMed, Google Scholar, and the Cochrane Library from inception to Oct 30, 2019 in order to identify relevant clinical trials for inclusion in this review. To avoid omitting relevant trials, we also searched conference summaries and reference lists from general reviews on mitophagy inducers treatment in AD. Two reviewers (WWW and XRZ) independently screened the titles, abstracts, and references from all identified reports.

The Medline (PubMed) search strategy was as follows:

(1) (Alzheimer’s disease [mh]) OR (Dementia) OR (Senile dementia) OR (Alzheimer Type Dementia) OR (ATD) OR (Alzheimer Sclerosis) OR (Alzheimer Syndrome) OR (Dementia, Presenile)
(2) (Mitophagy inducers) OR (mitophagy) OR (kinetin triphosphate) OR (KTP) OR (Pifithrin-a) OR (Deferiprone) OR (DFP) OR (Metformin) OR (phenanthroline) OR (Phen) OR (Ciclopirox olamine) OR (Nicotinamide riboside) OR (NR) OR (Nicotinamide) OR (NAM) OR (Nicotinamide mononucleotide) OR (NMN) OR (Resveratrol) OR (Fisetin) OR (P62/SQSTM1) OR (PMI) OR (Spermidine) OR (Urolithin A) OR (Actinonin) OR (Rapamycin) OR (Phenylhydrazones carbonyl cyanide m-chlorophenyl hydrazon) OR (CCCP) OR (phenylhydrazone) OR (FCCP) OR (dinitrophenol) OR (DNP) OR (BAM15) OR (Valinomycin) OR (Salinomycin) OR (Antimycin A) OR (Oligomycin) OR (Sodium selenite) OR (Diquat) OR (Retigeric acid B) OR (RAB) OR (Paraquat) OR (Rotenone) OR (1-methyl-4-phenylpyridinium) OR (MPP+) OR (6-hydroxyldopamine) OR (6-OHDA)
(3) (Clinical trial [mh]) OR (Clinical) OR (Random) OR (Placebo) OR (Blind) OR (Retraction of publication)
(4) (1) or (2) and (3)

Inclusion criteria

1 We included clinical trial that compared mitophagy inducers (no matter what kind of drugs) to placebo.
2 Subjects were required to have a clinical diagnosis of sporadic AD, of either sex and with mild, moderate or severe AD according to cognitive test.
3 Mitophagy inducers alone or in combination with other treatments compared with placebo alone or in combination with same treatments.

**Figure 3.**
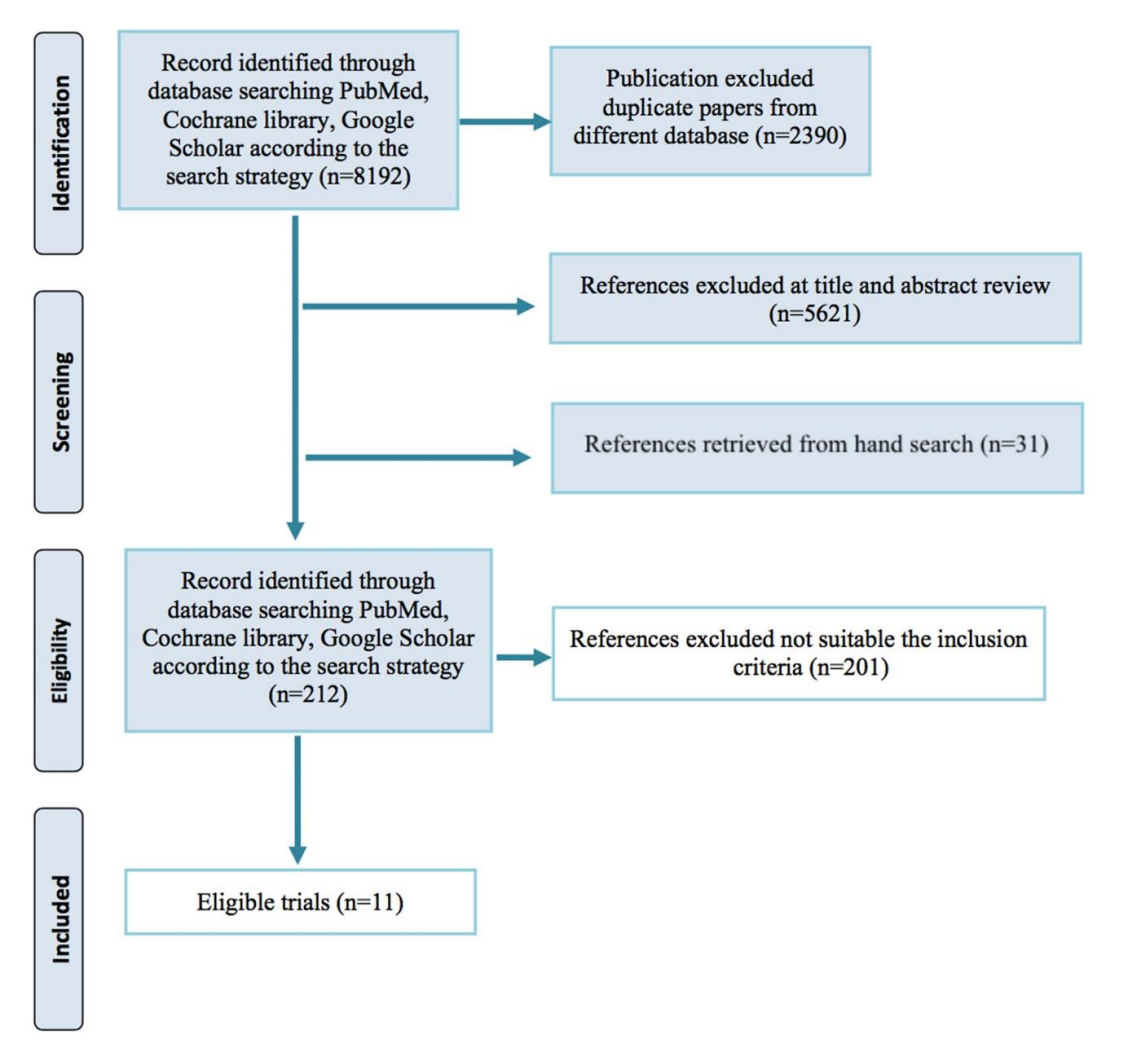
PRISMA 2009 flow diagram.

Finally, we identified 11 eligible clinical trial of mitophagy inducers for AD patients [50-61] (Figure 3). Eleven clinical trials with a total 466 AD patients were included in this review. The number of subjects in individual trial range from 17 to 119. Among them, Resveratrol and nicotinamide adenine dinucleotide (NADH) were utilized in 3 trials; Sodium selenite and Metformin in 2, Nicotinamide (NAM) was used in 1 trial. In terms of the dementia degree, eight studies subjects presented a mild to moderate [50-53, 55, 58, 59, 61]; two studies included mild AD subjects [54, 56]; one study did not report such information [60]. The mainly features and outcome results of the included clinical trials testing the effect of mitophagy inducers on AD *in vivo* were summarized in Table 2 and Table 3.

### Resveratrol versus placebo

Zhu *et al* [51] found RGM (resveratrol, glucose, and malate combination) treatment were similar on all of the screening variables. At 12 months duration, change scores on Alzheimer’s Disease Assessment Scale-cognitive subscale (ADAS-cog), Mini-Mental State Examination (MMSE), Alzheimer’s Disease Cooperative Study-Activities of Daily Living Scale (ADAS-ADL), or Neuropsychiatric Inventory (NPI) all showed less deterioration in the RGM than the control group. However, none of the change scores reached statistical significance (p > 0.05). Turner *et al* [58] reported resveratrol and its major metabolites were measurable in plasma and cerebrospinal fluid (CSF). CSF and plasma Aβ_40_ levels declined more in the placebo group than the resveratrol-treated group. Meanwhile, brain volume loss was increased by resveratrol treatment compared to placebo. Meanwhile, Moussa *et al* [53] showed that resveratrol decreased CSF MMP9, modulates neuro-inflammation, and induced adaptive immunity.

### NADH versus placebo

Demarin *et al* [61] reported that after 6 months of treatment, subjects treated with NADH (10 mg) showed no evidence of progressive cognitive deterioration and had significantly higher total scores on the MDRS compared with subjects treated with placebo (p < 0.05), such as better performance on verbal fluency (p = 0.019), visual-constructional ability (p = 0.038) and abstract verbal reasoning. There were no differences between groups in measures of attention, memory, or in clinician ratings of dementia severity. Rainer *et al* [59] performed a 3-month open-label study with oral 10 mg/day NADH with 25 patients with mild to moderate dementia of the AD, vascular, and found no evidence for any cognitive effect as defined by established psychometric tests. Finally, Birkmayer et al [60] demonstrated NADH improved the MMSE and global deterioration scale (GDS) scores compared to the placebo group, need more rigorously controlled studies to confirm in future.

### Metformin vs placebo

Twenty nondiabetic subjects with mild AD were randomized to receive metformin and placebo for 8 weeks in Koenig trial [54]. Metformin was associated with improved executive functioning, and trends suggested improvement in learning, memory and attention, as well as safe, well-tolerated, and measurable in CSF.

**Table 2:** Mainly features of the included studies testing the effect of mitophagy inducers on AD *in vivo*.

Study (year)	AD Diagnosis criteria and design	Mitophagy inducers	Demographics (Age; M/F)	Protocol	Outcome
Resveratrol vs placebo					
Moussa (2017)	NINCDS ADRDA (mild-moderate AD); Retrospective study	Resveratrol	Placebo: 73±8.2; 28M/27F; MMSE:20.7±4.3; ADAS-cog: 23.7±8.6; ADCS-ADL: 60.5±10.7; NPI: 11.1±11.6 Resveratrol: 69.8±7.7; 40M/24F; MMSE:20.2±4.4; ADAS-cog: 25.3±10.1; ADCS-ADL: 63.7±10.8; NPI: 7.5±7.9	Resveratrol (500 mg) or placebo orally once daily (with dose escalation by 500-mg increments every 13 weeks, ending with 1,000 mg twice daily) for 52 weeks	1. CSF/plasma biomarkers 2. MMSE, ADCS-ADL
Turner (2015)	NINCDS ADRDA (mild-moderate AD); Multicenter DB-RCT	Resveratrol			1. Plasma Ab40 and Ab42, CSF Aβ40, Aβ42, tau, and phospho-tau, and volumetric MRI; 2 MMSE, ADAS-cog, ADCS-ADL, CDR-SOB, NPI et al; 3. Safety and tolerability
Zhu (2019)	NINCDS ADRDA (mild-moderate AD); DB-RCT	RGM: 5mg Resveratrol, 5g glucose and 5g malate	Placebo group: 79.3±6.5; 8M/5F; MMSE:18.4±3.8; ADAS-cog: 29.2±8.9; ADCS-ADL: 46.6±7.6 RGM group: 80.5±8.6; 9M/7F; MMSE:18.1±4.9; ADAS-cog: 26.4±11.9; ADCS-ADL: 49.1±10.3	RGM twice a day in liquid form (15 mL) dissolved in unsweetened red grape juice for 1 year	1. ADAS-cog, MMSE, ADCS-ADL, NPI, ADCS-CGIC 2. Treatment adverse events
NADH vs placebo or base line					
Demarin (2004)	NINCDS ADRDA (mild-moderate AD); Multicenter DB-RCT	NADH	Of the 17 patients who completed the study, the age range was from 57 to 84 years; median age 77.5. The MMSE scores at baseline ranged from 12 to 24; with a median of 18.	Patients were randomly assigned to receive either NADH 5 mg, 2 tablets qd or matching placebo tablets for 6 months.	1. FOMT, HVLT, MMSE, MDRS, MTS acc, VF
Rainer (2000)	NINCDS ADRDA (mild-moderate AD); Open label one arm pilot study	NADH	NR	10 mg NADH with 25 patients per day for 12 weeks.	1. GDS, MMSE, ADAS-cog
Birkmayer (1996)	Based on cognitive test. Open label one arm pilot study	nicotinamide adenine dinucleotide (NADH)	NADH: 67.71 (33-84); 10M/7F; MMSE: 15.82 (16-24); GDS: 4.29 (2-6)	The total dosage was 10 mg NADH per day, which was given in the morning 30 minutes before the first meal for 12 weeks.	1. Cognitive test (MMSE, GDS)
Metformin vs placebo					
Koening (2017)	Cognitive tests and biomarkers (mild AD); DB-cross pilot study	Metformin	Placebo: 69.1±7.4; 5M/5F; MMSE:25±2.55; CDR-Global: 0.5±0; MoCA: 20.7±1.53; GDS: 1.4±1.43 Resveratrol: 71.1±6.57; 6M/4F; MMSE:26.9±1.46; CDR-Global: 0.8±0.78; MoCA: 19.5±0.7; GDS: 1±0.8	Metformin (500 mg/d) for 1 week, then increased by 500 mg per week until a maximum of 2000 mg/d for 8 weeks	1. Treatment adverse events 2. CSF indexes analysis 3. Functional imaging (ASL-MRI) 4. Cognition tests
Luchsinger (2016)	NR; Milde AD DB-RCT	Metformin	Placebo: 64.1±7.9; 16M/24F; 27.5% APOE4 carrier; ADAS-cog: 14.6±6.1 Resveratrol: 71.1±6.57; 6M/4F; 25% APOE4 carrier; ADAS-cog: 12.0±4.0	Metformin was titrated weekly from 500 mg once a day to 1000 mg twice a day over 4 weeks (total 12 weeks).	1. SRT, ADAS-cog, MMSE, CGIC-MCI 2. FDG-PET
Sodium selenite vs placebo					
Cardoso (2019)	NINCDS ADRDA (mild-moderate AD); Double-blind, randomized, placebo-controlled pilot study (DB-RCT)	Sodium selenate	Placebo group: 68.7±6.9; 3M/6F; MMSE:20.3±5.2; 66.7% APOE4 carrier Nutritional group: 73.4±5.5; 4M/4F; MMSE:19.5±2.4; 75% APOE4 carrier Supranutritional group: 69.5±8.3; 5M/14F; MMSE:20±3.5; 68.4% APOE4 carrier	Placebo: vehicle; Nutritional: 0.32 mg, tid, po; Supranutritional: 10 mg, tid, po For 24 weeks treatment	1. Selenium concentrations 2. Cognitive performance changes (MMSE; ADAS-cog; CFT; COWAT; OCL 3. Treatment adverse events
Malpas (2016)	NINCDS ADRDA (mild-moderate AD); Multicenter DB-RCT	VEL015 (Sodium Selenate)	Placebo: 71(61-81); 12M/8F; MMSE:20.3±5.2; 66.7% 65% APOE4 carrier; ADAS-cog: 22.08 VEL015: 73.4±5.5; 4M/4F; MMSE:19.5±2.4; 75% 70% APOE4 carrier; ADAS-cog: 19.68	VEL015 10 mg tid and placebo treatment for 24 weeks, and a 5-week post-treatment follow-up period.	1. Treatment adverse events 2. CSF indexes analysis 3. Structural MRI and PET 4.ADAS-cog, MMSE, COWAT,CFT, OCL et al
NA vs placebo					
Phelan (2017)	Modified NINCDS ADRDA (mild-moderate AD); DB-RCT	NAM^+^ precursor: Nicotinamide (NA)	Placebo group: 79.3±6.5; 12M/3F NA group: 80.5±8.6; 9M/7F	NA (1500 mg twice daily) or placebo for 24 weeks	1. ADAS-cog, CDR, MMSE, ADCS-ADL 2. Treatment adverse events

ADAS-cog: Alzheimer’s Disease Assessment Scale-cognitive; ADCS-ADL: Activities of Daily Living Scale; CDR-SOB: Clinical Dementia Rating-sum of boxes; NPI: and Neuropsychiatric Inventory; ASL: Arterial Spin Label; SRT: Selective Reminding Test; CSRT-MCI: Clinical Global Impression of Change for Mild Cognitive Impairment; FOMT: Fuld Object Memory recognition test; HVLT disc: Hopkins Verbal Learning Test discrimination index; MDRS: Mattis Dementia Rating Scale; MMSE: Mini Mental State Examination; MTS acc: Matching to Sample accuracy; VF, Verbal Fluency phonemic rule; M: male; F: female; NINCDS ADRDA: National Institute of Neurological and Communicative Diseases and Stroke/Alzheimer's Dis- ease and Related Disorders Association.

### Sodium selenite/ NAM vs placebo

Two trials concerning on the sodium selenite for AD patients. Malpas et al [55] found VEL015 (sodium selenate) at doses up to 30 mg per day for 24 weeks was safe and well-tolerated in patients with AD, however no difference in the fields of MMSE, ADAS-cog et al cognition tests, which is consistent with Cardoso et al trial no significant benefit on cognitive performance by sodium selenite [50]. In the end, NAM also failed to demonstrate improving cognitive function in subjects with mild to moderate AD over 24 weeks. The lack of efficacy of NAM may have been due to several contributing factors including a low sample size, inclusion of subjects with moderate AD and so on [52]. Based on aforementioned trials, most evidence does not support use of the mitophagy inducers for cognitive protection in persons with AD, the results have not been clinically impressive. However, in terms of safety consideration, such compounds are well tolerated. Until now, we need to find more promising mitophagy inducers and carried out large, high quality randomized controlled trials (RCTs) on AD. Notably, there are still some issues should be considered. 1) The compounds listed as mitophagy activators have numerous other effects in many tissues and in most cases the true 'target' is still not clear; 2) Most also activate other autophagy pathways (e.g. Metformin and Resveratrol)-there is no specificity to mitophagy; 3) Most of these compounds had no beneficial effect in human trials. Thus, we need to develop more rigorously and specificity mitophagy inducers that can restore defective mitophagy.

**Table 3:** Summarize the mainly effects of the mitophagy inducers in the included studies.

Study (year)	Summarize the effects of the mitophagy inducers in outcome
Resveratrol vs placebo	
Moussa (2017)	RGM (resveratrol, glucose, and malate combination) treatment were similar on all of the screening variables. At 12 months treatment duration, change scores on ADAS-cog, MMSE, ADAS-ADL, or NPI fields all showed less deterioration in the RGM than the control group. However, none of the change scores reached statistical significance (p > 0.05).
Turner (2015)	Resveratrol and its major metabolites were measurable in plasma and CSF. The most common adverse events were nausea, diarrhea, and weight loss. CSF Aβ_40_ and plasma Aβ_40_ levels declined more in the placebo group than the resveratrol-treated group, resulting in a significant difference at week 52. Brain volume loss was increased by resveratrol treatment compared to placebo.
Zhu (2019)	Compared to the placebo-treated group, at 52 weeks, resveratrol markedly reduced CSF MMP9 and increased macrophage-derived chemokine (MDC), interleukin (IL)-4, and fibroblast growth factor (FGF)-2. Compared to baseline, resveratrol increased plasma MMP10 and decreased IL-12 P40, IL12 P70, and RANTES. In this subset analysis, resveratrol treatment attenuated declines in MMSE scores, change in ADCS-ADL scores, and CSF Aβ_42_ levels during the 52-week trial, but did not alter tau levels.
NADH vs placebo or base line	
Demarin (2004)	After 6 months of treatment, subjects treated with NADH (10 mg) showed no evidence of progressive cognitive deterioration and had significantly higher total scores on the MDRS compared with subjects treated with placebo (p < 0.05), such as better performance on verbal fluency (p = 0.019), visual-constructional ability (p = 0.038) and abstract verbal reasoning.
Rainer (2000)	No clinically relevant changes versus baseline were seen in the GDS, the cognitive parameters, or any of the three subscores of the ADAS-Cog that capture memory, orientation and language in the group as a whole. Moreover, none of the minute changes that where observed in these parameters achieved statistical significance or indicated a statistical trend.
Birkmayer (1996)	NADH improved the MMSE and GDS scores compared to the placebo group, need more rigorously controlled studies to confirm in future.
Metformin vs placebo	
Koening (2017)	Metformin was found to be safe, well-tolerated, and measureable in CSF. Metformin was associated with improved executive functioning, and trends suggested improvement in learning/memory and attention. No significant changes in CBF were observed, though post-hoc completer analyses suggested an increase in orbitofrontal CBF with metformin exposure.
Luchsinger (2016)	Metformin could not be tolerated by 7.5% of participants. There were no serious adverse events related to metformin. The 7.5% of persons who did not tolerate metformin reported gastrointestinal symptoms. After adjusting for baseline ADAS-cog, changes in total recall of the SRT favored the metformin group. Differences for other outcomes were not significant.
Sodium selenite vs placebo	
Cardoso (2019)	Supranutritional selenium supplementation was well tolerated and yielded a significant increase in CSF selenium. Reclassifying subjects as either responsive or non-responsive based on elevation in CSF selenium concentrations revealed that responsive group did not difference in Mini-Mental Status Examination (MMSE) as non-responsive group.
Malpas (2016)	VEL015 (sodium selenate) at doses up to 30 mg per day for 24 weeks was safe and well-tolerated in patients with AD, however no difference in the fields of MMSE, ADAS-cog et al cognition tests.
NA vs placebo	
Phelan (2017)	There were no significant effects of NA on the primary or secondary endpoints. A mild effect of low compliance was observed on word recall and command tasks. There were no differences in adverse events experienced by NA- and placebo-treated groups.

ADAS-cog: Alzheimer’s Disease Assessment Scale-cognitive; ADCS Activities of Daily Living Scale (ADCS-ADL); and Neuropsychiatric Inventory (NPI); MDRS: Mattis Dementia Rating Scale; MMSE: Mini Mental State Examination; Cerebral blood flow: CBF; GDS: global deterioration scale.

## Developing lead candidate mitophagy inducers-deep learning

Deep learning is a form of machine learning techniques that enables computational models, which are constituted of multiple processing layers by using the backpropagation algorithm to learn plentiful of data with multiple levels of abstraction [62]. The method has made impressive recent advance in application and dramatically improved the “state-of-the-art” in speech recognition, object detection, visual object recognition and many other domains such as drug discovery and genomics et al [63, 64]. In addition, deep learning has also been broadly covered in the biomedical fields and impact a few key areas of medicine. Among them, computer vision mainly focuses on medical imaging, and natural language processing electronic health record data. Therefore, reinforcement learning is utilized in the context of robotic-assisted surgery [65].

**Figure 4.**
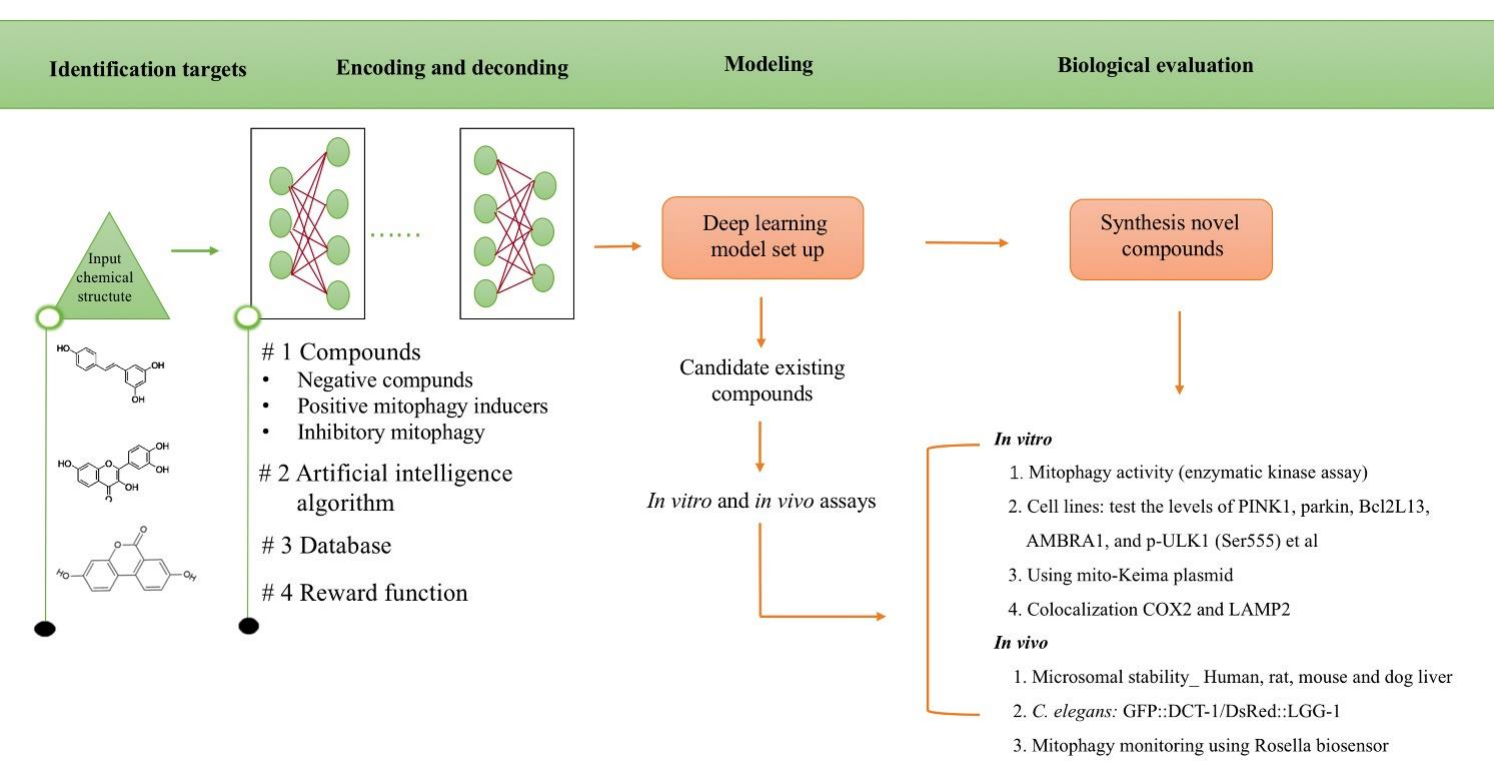
Deep learning for mitophagy inducers development: The general workflow for the design of lead candidates using deep learning model. The workflow comprises identification targets, Encoding and decoding, establish models and Biological evaluation *in vitro* and *in vivo*.

Recently, Alex Zhavoronkov and his colleagues have developed a deep generative model, generative tensorial reinforcement learning (GENTRL) for *de novo* small-molecule design. They used GENTRL model to discover one lead potent inhibitor of discoidin domain receptor 1 (DDR1) in less than two months as well as only for a fraction of the cost associated to the traditional drug discovery process [66], indicating this approach is promising to provide rapid and efficient drug screening. Therefore, in future, we can use this technology to discovery potential mitophagy inducers. Figure 4 shows the general workflow for the design of lead candidates mitophagy inducers using deep learning, as well as following biological evaluation to test the mitophagy levels *in vitro* and *in vivo* levels [67]. Although deep learning has displayed impressive potential advantage in rapid identification drugs, a series of outstanding issues remain [68]. First is the challenge of how to figure out deep learning models that best optimize augment and complement human experience in making medical decisions, such as chemical structures interpretation. Next, how to avoid biases in training sets and how to interpret predictions. Finally, there is essential for iterative experimentation, in which deep learning predictions can be tested and more accurate by biological laboratory evaluation or by formal clinical assessment. However, like any scientific advance of importance, these methods unfold numerous new questions as they answer. Undoubtedly, there are much exciting and challenging works have to be done, requiring the continued close interaction between computer science, pharmacology and biological medicine [69].

## Future perspective

AD patients in whom brain Aβ deposition and p-tau were virtually decreased by anti-Aβ/tau therapies and however did not show any cognitive benefit or serious toxicity owing to off-target effects in clinical trials over the past decades [13]. An alternative promising option for AD therapeutics is to address the dysfunction of mitophagy for developing AD. One outstanding issue should be emphasis is different theories such as “amyloid plaques, NFT, neuroinflammation, mitochondrial dysfunction, and comprised mitophagy” result in AD etiologies that interaction with each other [70]. The ‘chicken and egg’ relationships between different hallmarks of AD need to be established in future, and specific therapy should be directed target the reason of the neuronal insult and not the host response. Meanwhile, we can pick up some clues from other diseases of aging, including heart disease, cancers, and hypertension et al that combination administrations based on different theories are essential and reasonable for AD patients.

Major milestones in the mitophagy field have been achieved in the last decade. Nevertheless, important questions remain regarding the *in vivo* role of mitophagy components, the spatiotemporal regulation of mitophagy within distinct physiological and pathological contexts and the complex interplay between different mitophagy pathways, as well the need to identify novel chemical probes that can be used to understand the process of mitophagy and correct defects [49]. Combining *in vivo* mitophagy imaging systems with disease animal models could help to unravel disease etiology and progression and contribute to translational research. Chemical-induced mitophagy stimulation should be further evaluated *in vivo* in different cell types and tissues. Identifying mitophagy modulators may lead to therapeutic intervention strategies targeting mitochondrial-associated pathologies and provide critical insights with broad relevance to human health and quality of life [31]. In the real word, triggering mitophagy through a pharmacologically induced acute depolarization has limitations (refer to toxic mitophagy inducers), highlighting the necessary for vicarious means to regulate the process and, eventually, discover non-toxic inducers for therapeutic purposes [71]. Interestingly, NAD^+^ precursors, including NMN, NR, are promising drug candidates in view of their natural existence in the human body and their safety and efficiency in preclinical trials. Notably, we can encode and decoding the exist non-toxic mitophagy inducer structures through deep learning and identify some potential promising compounds (Fig. 3).

## Conclusions

We systematic review the current status of mitophagy modulators and analyzed their relevant mechanisms, commenting on their advantages, limitations and current applications in clinical trials for AD patients. Finally, we describe how deep learning may be a promising method to rapid and efficient discovery of mitophagy inducers as well as general guidance for the workflow of the process.
